# From cholera to enterotoxigenic *Escherichia coli* (ETEC) vaccine development

**Published:** 2011-02

**Authors:** Ann-Mari Svennerholm

**Affiliations:** *WHO Collaborating Centre for Research on ETEC & Gothenburg University Vaccine Research Institute (GUVAX), Department of Microbiology & Immunology, Institute of Biomedicine, University of Gothenburg, Gothenburg, Sweden*

**Keywords:** B subunit, cholera, colonization factors, ETEC, ileal loops, oral vaccines

## Abstract

It was shown earlier that immune responses against cholera toxin (CT) as well as *Vibrio cholerae* lipopolysaccharide (LPS) or whole bacterial cells (WC) were protective and that these different antibody specificities co-operated synergistically for protection against experimental cholera. Similarly, antibodies against the heat-labile toxin (LT) and major colonization factors (CFs) of enterotoxingenic *Escherichia coli* (ETEC) co-operated synergistically for protection against LT-producing ETEC expressing homologous CFs. Studies in humans revealed that repeated oral antigen administration was optimal in inducing intestinal immune responses. Based on these findings oral inactivated vaccines consisting of toxin antigen and whole cells, *i.e*. the licensed recombinant cholera B subunit (rCTB)-WC cholera vaccine Dukoral®, and candidate ETEC vaccines have been developed. In different trials the rCTB-WC cholera vaccine has provided very high (85-100%) short term protection, which was significantly higher than that induced by the WC component alone, whereas rCTB-WC and WC alone provided comparable (50-60%), long term protection. An oral ETEC vaccine consisting of rCTB and formalin-inactivated *E. coli* bacteria expressing major CFs was shown to be safe and immunogenic in adults and children in different countries. The vaccine also induced significant protection against non-mild ETEC diarrhoea, *i.e*. diarrhoea interfering with daily activity in American travellers but not against ETEC diarrhoea in young children in Egypt. Against this background, a modified ETEC vaccine consisting of recombinant *E. coli* strains overexpressing the major CFs and a more LT like hybrid toxoid (LCTB*A*) has been developed. This vaccine will be tested soon alone and together with a mucosal adjuvant, *i.e*. dmLT, in clinical trials.

## Introduction

*Vibrio cholerae* O1 and enterotoxigenic *Escherichia coli* (ETEC) are two major bacterial pathogens responsible for a high proportion of diarrhoeal disease and death in adults and children in many countries in Africa and Asia. The pathogenic mechanisms of these two bacteria are very similar in that they cause disease by colonizing the epithelium of the small bowel and producing enterotoxins responsible for the diarrhoeal fluid induced. The cholera toxin (CT) produced by *V. cholerae* O1 and the heat-labile enterotoxin (LT) of ETEC are structurally, functionally and immunologically closely related. However, CT is secreted extracellularly, while LT is trapped in the periplasm of the bacterial cells, but released after cell lysis. The small polypeptide heat-stable enterotoxin (ST) is an additional virulence factor of ETEC and may be present in strains with or without LT^1^.

Based on the detailed elucidation of the pathogenic mechanisms of cholera and ETEC^1^, we have postulated that protection against the causative organisms should be directed not only against colonization of the bacteria, but also against the toxin action. Furthermore, we deduced that immunity in the small intestine was of prime importance for preventing disease. Hence, efforts in our laboratory to develop effective vaccines against cholera and ETEC diarrhoea have been focused on the identification of major protective antigens preventing binding of the bacteria in the intestine, suitable toxoids and optimal ways of inducing intestinal immune responses.

## Development of a cholera vaccine

**Fig. 1:**
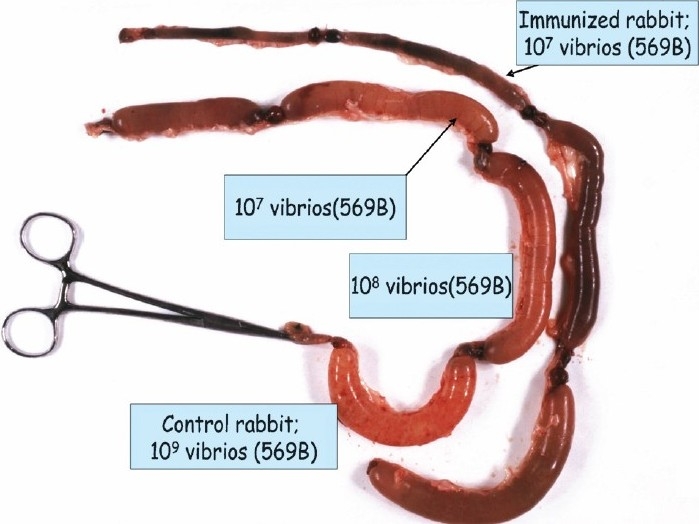
Use of rabbit ileal loop technique for identification of protective antigens against cholera as a basis for vaccine development.

To evaluate the possible protective roles of antibacterial and antitoxic immunities in cholera we made use of the elegant rabbit ileal loop technique developed by De^2^ and modified the assay slightly to allow determination of the protective efficacy of different cholera antigens^3^. This was done by challenging ileal loops of immunized young New Zealand white rabbits with graded doses of fully virulent *V. cholerae* bacteria (strain 569B), and determination of the dose of bacteria causing 50 per cent fluid accumulation (ED_50_) in the loops of the animals sacrificed on the following morning. By comparing the ED_50_ of the challenge organisms in animals immunized with different antigens, *i.e*. cholera toxin (CT), *V. cholerae* lipopolysaccharide (LPS), and whole cell vibrios (WCV), and control (phosphate buffered saline, PBS-injected) rabbits we could determine the so called protection factor, *i.e*. the ratio between ED_50_ in immunized and control animals (Fig 1).

**Fig. 2:**
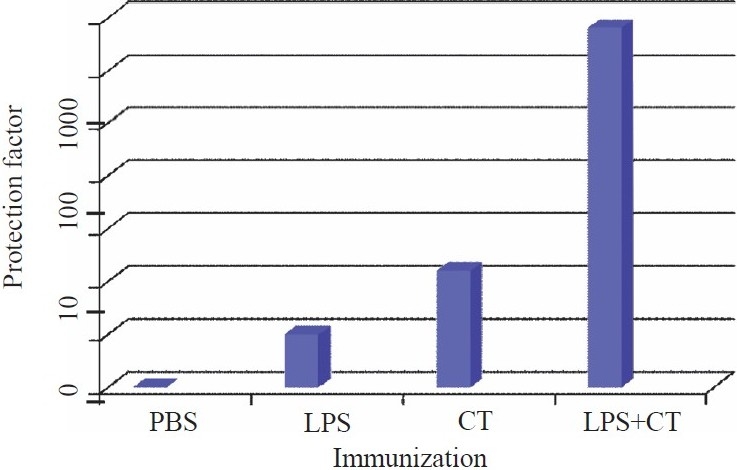
Rabbits subcutaneously immunized with 2 doses of *V. cholerae* LPS (1.25 mg/dose), CT (15 μg/dose) or a combination of the same doses of LPS and CT were challenged with graded doses of live vibrios, and ED_50_ for immunized rabbits were compared with those of PBS injected rabbits. Protection factors are indicated on y-axis.

By such experiments we could show that CT and LPS were both protective, *i.e*. considerably higher doses of vibrios were required for ED_50_ in CT and LPS immunized rabbits, respectively than in control animals. Furthermore, immunization with LPS and CT resulted in a protective effect which exceeded the additive effects induced by each antigen alone, *i.e*. the two types of antigens co-operated synergistically for protection against experimental cholera^3^ (Fig 2).

Using the same rabbit ileal loop technique, we demonstrated that the B subunit component of CT, *i.e*. CTB, was equally effective as whole CT in inducing protection against live *Vibrio* challenge and that CTB in combination with LPS or whole inactivated WCV also provided synergistic protection against experimental cholera^4^.

In subsequent human volunteers studies both in Sweden and in Bangladesh, we could show that repeated oral, rather than parenteral or different combinations of oral and parenteral, immunizations with cholera antigens were superior in eliciting immune responses locally in the intestine using the so called intestinal lavage technique^5^. Indeed, a single oral dose with 2.5 mg of CTB was equally effective as clinical cholera in eliciting antitoxic IgA responses locally in the intestine of adult Bangladeshis, and two oral doses of 5×10^10^ killed WCV induced comparable antibacterial immune responses in intestine as in clinical cholera^5^. These different findings were the basis for the design of the oral CTB - whole cell (WC) cholera vaccine. This vaccine has been extensively tested and shown to provide very high short term protection (85-100%), *e.g*. in studies in different age groups in Bangladesh and Peru, and prolonged protective efficacy (50-60%) for 2-3 years in endemic populations. The WC component alone has provided comparable long term protection, but was significantly less effective than the combined CTB-WC vaccine in inducing short term protection^6^ (Table I).

**Table I:** Protection afforded by oral inactivated CTB- whole cell (WC) and WC alone cholera vaccines in studies in different countries

Vaccine	Countries	No. of subjects; age group (yr)	Protective efficacy (%)
CTB-WC O1	Bangladesh, 1985; re-analysis- 2005	31, 200;≥2	85, 6 months; 50-60, 3 yr herd protection
rCTB-WC O1	Peru, 1994	1,426; 17-65	85, 3-6 months
(Dukoral®)	Mozambique, 2005	21,818; > 1	78, 1 yr
WC O1	Bangladesh,1985;	31,150;≥.2	60, 3 yr
	re-analysis 2005		herd protection
	Vietnam, 1992	67,395; >1	66, 1 yr
	India (Kolkata), 2008	69,000;≥2	68, 1 yr
CTB, B subunit of cholera toxin			

## Development of oral inactivated ETEC vaccines

Since ETEC is still the most common cause of diarrhoea in the developing world resulting in approximately 20 per cent of all diarrhoeal episodes in children in these areas, and the most frequent cause of diarrhoea in travellers^7^, we are intensively working on the development of an ETEC vaccine. Although there is no effective ETEC vaccine available yet, there is strong evidence to support that such a vaccine may be developed. Thus, in regions of the world where ETEC is highly endemic there is a decline in ETEC diarrhoeal incidence with increasing age with peaks observed in the age groups 6-18 months^7^,^8^, whereas no such age-related association is evident in short time visitors to endemic areas^7^. However, the incidence of ETEC rapidly decreases also in persons from industrialized countries during prolonged stay in ETEC endemic areas. These observations strongly suggest that effective immunity may develop after repeated infections and, as a consequence protection by way of an effective ETEC vaccine is achievable. The design of such a vaccine should be based on the knowledge of mechanisms of disease and immunity in ETEC infections. Based on the similarities of the pathogenic mechanisms between *V. cholerae* and ETEC, we have applied similar approaches as we used for cholera to identify protective ETEC antigens and optimal modes of eliciting intestinal immune responses.

## Virulence factors and identification of protective antigens in ETEC

The major virulence mechanisms in ETEC include production of LT and/or ST^1^,^7^. Immunity against LT is predominantly directed against the B subunit component of LT (LTB) which is 80 per cent homologous with CTB^1^. ST, which is a very small molecular weight peptide consisting of 18 or 19 amino acids, is not antigenic unless coupled to a carrier protein^1^,^9^. Hence, immune responses to ST are not induced after infection with ST producing ETEC. The relative proportion of strains producing LT alone, ST alone or LT/ST varies from one geographic area to another; overall 30-50 per cent of clinical ETEC isolates seem to produce ST only^1^,^7^,^9^. ETEC is a very heterogeneous group of bacteria and more than 100 different O-serogroups of *E. coli* having been identified among clinical ETEC isolates^10^. In addition, rough strains which are non-typeable with regard to O-antigen are not uncommon^10^. Although there are certain ETEC serogroups which are more prevalent than others, there are large geographical differences.

Other important virulence factors in ETEC include production of one or more colonization factors (CFs), which usually are fimbriae^10^,^11^. More than 25 CFs have been recognized on human ETEC so far, and additional ones are likely to be recognized^11^. The CFs promote colonization of ETEC in the small bowel, thus allowing expression of the toxins in close proximity to the intestinal epithelium. Of the wide range of CFs, the most commonly present on clinical isolates include CFA/I, CS1, CS2, CS3, CS4, CS5, CS6, and in some studies also CS7, CS14, CS17 and CS21^7^,^9^. Several of these CFs may be expressed on the same bacteria, *e.g*., CFA/II strains may express CS1+CS3 or CS2+CS3 or CS3 alone and CFA/IV strains may express CS4+CS6 or CS5+CS6, although an increasing number of strains express CS6 alone. The different CFs have been found on ETEC in varying frequencies (50-80%) in different geographic areas, during different seasons and in different categories of patients^7^. Some of the better characterized CFs are related, *i.e*. the colonization factor I-like group (including CFA/I, CS1, CS2, CS4, CS14, CS17, CS22 and PCFO71) and the coli surface 5-like group (with CS5, CS7, CS18, CS20)^12^. Strains expressing CFs within these groups have been shown to induce substantial immune responses not only against the homologous, but also against other CFs within the respective groups^7^. Most of the CFs are composed of up to 1000 identical structural subunits and several of the CFs also express distinct tip proteins^12^.

**Fig. 3:**
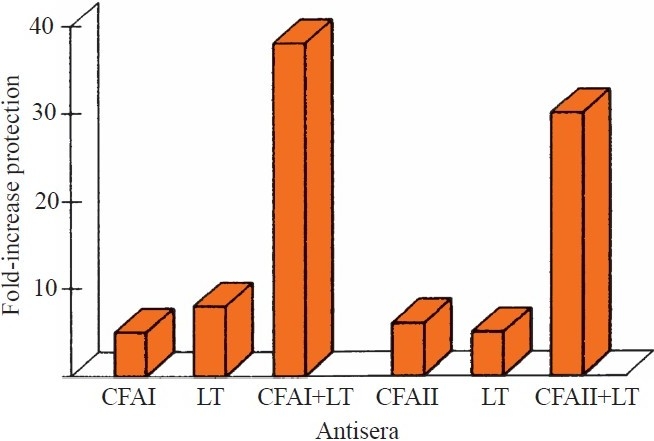
Protection afforded by rabbit antisera against CFA/I, CFA/II (CS1+CS3) and LT against challenge with ETEC expressing homologous colonization factors (CFs) and LT in rabbit small bowel loops.

Using similar technology as described for cholera, we applied a modified rabbit small bowel loop technique to evaluate the capacity of specific antibodies against these putative protective ETEC antigens to prevent experimental ETEC infection in passive protection studies. By ligating between twenty five and thirty 4-5 cm long loops in each rabbit and testing different concentrations of the challenge bacteria in combination with a certain dilution of specific antiserum, different antibody specificities could be tested against one or more challenge ETEC strains in the same animal. In initial experiments, antisera against LT as well as purified *E. coli* LPS were shown to provide protection against challenge with LT-producing O group homologous ETEC strains^13^. Subsequently, we tested the protective effect of anti-LT antibodies in combination with antisera against CFA/I or CFA/II (CS1+CS3) for protection against LT- producing CFA/I or CFA/II (CS1+CS3) positive ETEC strains. Indeed, the anti-CF sera provided significant protection against challenge strains expressing homologous CF antigens. Further, anti-LT and anti-CF sera also co-operated synergistically for protection against corresponding challenge strains^13^ (Fig. 3).

Mixtures of challenge bacteria with monoclonal antibodies against different CF antigens, *e.g*., CS1,CS2 and CS3 also resulted in increased ED_50_ of challenge strains expressing the corresponding CFs, but not against ETEC expressing heterologous CS antigens. These results were corroborated in subsequent experiments using the rabbit non-ligated intestine (RITARD) model. Thus, infection with ETEC expressing certain CFs provided highly significant protection against re-infection with fully virulent ETEC expressing the homologous, but not heterogonous ETEC CFs^14^. Further support for a protective effect of ETEC CFs is provided by our recent findings from a birth cohort study in Bangladesh that re-infections with ETEC expressing homologous CFs are rare, whereas re-infections with LT producing strains are rather common^8^.

## Development of an oral inactivated ETEC vaccine

Based on the identification of CFs as key protective ETEC antigens, our approach to develop a vaccine has been to prepare killed ETEC that express the most important CFs in immunogenic form on the bacterial surface^9^. Inactivation of the bacteria may be achieved by treatment with formalin^9^, which has resulted in killing of the bacteria without significant loss in antigenicity of different CFs and O-antigens. Thus, CFs on ETEC inactivated by mild formalin-treatment have been shown to be more stable than purified CFs in the gastrointestinal milieu as well as to retain their immunogenicity, fimbrial structure and capacity to bind to eukaryotic cells. To provide enhanced protection against LT producing ETEC, the inactivated bacteria may be combined with an appropriate LT toxoid. The vaccine should also be given by the oral or gastro-enteral route to induce optimal immune responses locally in the intestine^9^.

Against this background we developed an ETEC vaccine consisting of a combination of recombinantly produced CTB (rCTB) and formalin-inactivated ETEC bacteria expressing CFA/I and CS1-CS5 as well as some of the most prevalent ETEC O-antigens^9^. This rCTB-CF ETEC vaccine was shown to be safe and to give rise to significant IgA immune responses locally in the intestine in a majority, 70-90 per cent, of Swedish vaccines^15^. Phase I and II trials in adult volunteers in Sweden, Bangladesh and Egypt revealed that the vaccine was well tolerated and gave rise to mucosal immune responses, *i.e*., immune responses in intestine or peripheral blood antibody-secreting cells (ASCs) against the different vaccine CFs in 70-100 per cent of the vaccines^7^,^16^,^17^. Furthermore, the vaccine was shown to induce comparable immune responses against the CFs and LT locally in the intestine as in clinical ETEC disease^18^. Safety and immunogenicity clinical trials conducted in children in developing countries^9^,^19^,^20^, showed that the vaccine was well tolerated, except in the youngest infants, and almost equally immunogenic in children as in the adults. Since increased frequency of vomiting was observed in children 6-17 months of age in Bangladesh^20^, a dose finding study was initiated. This study showed that a quarter of a full dose of rCTB-CF ETEC vaccine was safe also in young infants and elicited immune responses both against LT and the CFs in the vaccinees.

The protective efficacy of the rCTB-CF ETEC vaccine was assessed in two larger placebo-controlled Phase III trials in American travellers going to Mexico and Guatemala. The first study, encompassing nearly 700 volunteers^21^, did not meet primary endpoints but the vaccine provided significant protection (PE 77%; *P*=0.039) against non-mild ETEC diarrhoeal illness, defined as symptoms that interfered with the travellers’ daily activities. However, no significant protection was observed against ETEC diarrhoea of any severity, including mild cases^21^. A subsequent equally-sized trial in the same setting also revealed that the vaccine protected against more severe symptoms in those volunteers in which vaccine take could be documented (Bourgeois *et al*, personal communication)^22^.

The only paediatric study to assess efficacy of the rCTB-CF ETEC vaccine was undertaken in rural Egypt with 350 children of 6-18 months old (Savarino *et al*, personal communication)^22^. In that placebo-controlled trial with active surveillance, through semi-weekly household visits and cultures of faecal specimens from children with diarrhoea, no significant protection was induced by the vaccine (protective effect, PE=20%). In part, this could be due the fact to that most cases were relatively mild, which is known to result in lower protective efficacies as compared to when passive surveillance was performed and protection against moderate to severe dehydration determined. It may also be explained by the finding that the young children participating in the Egyptian trial seemed to respond less well immunologically to the vaccine than similarly immunized older children and adults in the same setting as well as in Swedish and American adults (unpublished data). This finding is in agreement with observations for several other oral vaccines, *e.g*., poliovirus and rotavirus vaccines, which all were shown to be considerably less immunogenic in infants and young children in the developing world than in adults in industrialized countries^23^.

## Further development of the rCTB-CF ETEC vaccine

Based on the results of testing the rCTB-CF ETEC vaccine in children in Egypt, studies to improve its efficacy are in progress. These efforts include increasing the amounts of protective antigens in the vaccine, in particular the CFs on the bacterial surface^9^. By using recombinant technology, CFA/I could be expressed in considerably higher quantities on the surface of *E. coli* K12 bacteria than on previous vaccine strains, as determined by different immunoassays and immunoelectron microscopy^24^. Indeed, the recombinant *E. coli* strain expressed up to 10-fold higher levels of CFA/I fimbriae compared to the CFA/I positive strain that was used in the original rCTB-CF ETEC vaccine. The latter strain had previously been shown to be among the highest natural producers of the CFA/I fimbriae among >100 tested wild type ETEC strains. Mice orally immunized with formalin-killed bacteria of the CFA/I overexpressing *E. coli* strain induced significantly higher serum IgA antibody responses compared to the old vaccine strain^24^. Using a similar approach, other prevalent ETEC CFs have been overexpressed on the surface of *E. coli* K12 or non-toxigenic ETEC. For example, a non-toxigenic *E. coli* strain that overexpresses the non-fimbrial CS6 protein in up to 20-fold higher quantities than previous vaccine strains has been developed^25^. Other *E. coli* strains, including non-toxic ETEC strains that over-express CS2, CS3, CS4 and CS5 have recently been constructed^26^. Alternative methods have also been developed that allow inactivation of CS6 positive strains with retained CS6 antigenicity, since this protein is sensitive to formalin treatment.

Other efforts to improve the efficacy of the rCTB-CF ETEC vaccines include usage of an alternative LT toxoid, *e.g*., a more LT like toxoid, *i.e*., a hybrid LTB/CTB (LCTBA) toxoid^26^. This hybrid protein has been shown to be safe and provide better LT neutralizing immune responses than CTB in experimental animals^27^ (unpublished data). The efforts also include evaluation of the capacity of different putative mucosal adjuvants, in particular a double-mutated LT (dm LT) molecule^28^. Recent studies in our laboratory have shown that dmLT was safe with strong adjuvant activity on CF-producing *E. coli* strains in experimental animals (Holmgren J, *et al*, unpublished observation). Studies are also planned to administer the ETEC vaccine by different routes, *e.g*., by the simple sublingual route, which has recently been shown to be very efficient in inducing intestinal immune responses^29^.

Based on these considerations, a more definitive formulation of the oral inactivated ETEC vaccine has been developed and production under good manufacturing practice (GMP) conditions initiated to allow clinical trials of safety and immunogenicity of the new candidate vaccine (Table II). Different clinical trials are planned, initially in Sweden, to evaluate the capacity of *E. coli* overexpressing CFs to induce significantly higher systemic and mucosal anti-CF immune responses as compared to previous corresponding vaccine strains. These studies will also include an evaluation whether the LCTB*A* hybrid protein may induce significantly higher immune responses against LT than CTB provided results in Sweden appear to be promising and the vaccine will subsequently be tested for safety and immunogenicity in the most important target group, *i.e*. young children in ETEC endemic countries.

**Table II:** Next generation oral inactivated ETEC vaccine

Strategy
Four *E.coli* strains overexpressing the most important CFs Strains over-expressing CFA/I, CS3, CS5 and CS6 (*i.e*. 5-20-fold higher levels of CFs per 10^10^ bacteria than the best clinical isolates
LCTB*A* hybrid protein replacing rCTB
Usage of dmLT or alternative mucosal adjuvant if preclinical and clinical studies show enhancement of mucosal immune responses
CFs, colonization factors; CFA, colonization factor antigen; CS, coli surface antigen; dmLT, double-mutated
